# Protein-encapsulated fluorogenic probes for the selective detection of endogenous O-GlcNAcase (OGA)

**DOI:** 10.1039/d5sc06843f

**Published:** 2026-02-17

**Authors:** Yuan-Hao Wu, Chen Guo, Zi-Ru Ye, Xi-Le Hu, Tony D. James, Jia Li, Xiao-Peng He

**Affiliations:** a Key Laboratory for Advanced Materials and Joint International Research Laboratory of Precision Chemistry and Molecular Engineering, Feringa Nobel Prize Scientist Joint Research Center, School of Chemistry and Molecular Engineering, East China University of Science and Technology 130 Meilong Rd Shanghai 200237 China xphe@ecust.edu.cn; b The International Cooperation Laboratory on Signal Transduction, Eastern Hepatobiliary Surgery Hospital, National Center for Liver Cancer Shanghai 200438 China; c National Center for Drug Screening, State Key Laboratory of Drug Research, Shanghai Institute of Materia Medica, Chinese Academy of Sciences Shanghai 201203 China jli@simm.ac.cn; d Department of Chemistry, University of Bath Bath BA2 7AY UK t.d.james@bath.ac.uk; e School of Chemistry and Chemical Engineering, Henan Normal University Xinxiang 453007 China

## Abstract

Ever-increasing evidence confirms the role of O-GlcNAcase (OGA) in mediating cell growth and development as well as pathology and underscores the importance of developing sensitive and selective chemical tools for the study of OGA biology. Here, based on our previously developed protein-encapsulation strategy, we designed and synthesized a series of fluorogenic probes based on resorufin, and assembled their composites with human serum albumin (HSA) for the selective detection of endogenous OGA activity in live cells. We show that host–guest self-assembly with HSA significantly enhances the OGA sensitivity of the probes in aqueous solution and cells. The structure of the complex of a glycoprobe and HSA was resolved by small-angle X-ray scattering. We demonstrate that the replacement of the acetyl group in GlcNAc with a propionyl group results in selectivity for OGA over hexosaminidases (HEX) that unselectively hydrolyze hexosamines. This allows us to differentiate between two cell lines with different endogenous OGA and HEX expression levels, and selectively detect OGA activity in live cells.

## Abstract 2

A human serum albumin (HSA) encapsulated fluorogenic glycoprobe was developed for the selective detection of *O*-GlcNAcase (OGA) over hexosaminidase (HEX) in cell lysates and live cells.

## Graphical abstract

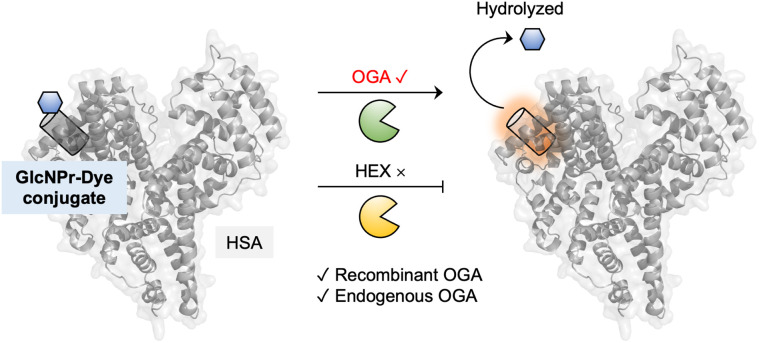

## Introduction

Mounting evidence has uncovered the important role of O-linked *N*-acetylglucosaminylation (O-GlcNAcylation) in mediating cellular actions including cell growth, division, differentiation and development.^1^ The malfunctioning of this biochemical process is also correlated with a number of pathological processes that can ultimately lead to human diseases such as cancer and neurodegenerative disorders.^2,3^ Protein O-GlcNAcylation is dynamically tailored by the reverse action of O-GlcNAc transferase (OGT) and O-GlcNAcase (OGA).^4^ Chemical probes developed for OGT mainly rely on a metabolic labelling strategy using non-natural GlcNAc derivatives (*e.g.*, azide or alkyne-modified GlcNAc probes) in combination with a biorthogonal tag.^5,6^ This strategy has led to innovative observations indicating that OGT activity is involved in modulating a number of signalling pathways relevant to cellular proteostasis, cardiac function and cancer.^7,8^

On the other hand, OGA removes GlcNAc from the serine/threonine residues of proteins. Previous investigations have shown that OGA is implicated in a series of physiological and disease–relevant processes.^9–11^ In addition, a recent study suggests that one role of OGA is to promote cancer cell malignancy by modulating the O-GlcNAcylation level on PDZ and LIM domain protein 7 (PDLI7).^12^ Conventional approaches to detect OGA activity have mainly relied on indirect analysis of the overall O-GlcNAcylation level using immunoblotting. This protocol is easily interfered with by fluctuations in OGT activity as well as the non-specific binding of *anti*-O-GlcNAc antibodies. As a consequence, colorimetric and fluorogenic substrates for OGA have been developed. For example, bis-acetal-based substrates have been developed as tunable fluorescence-quenched probes for OGA.^13^ By tuning the intramolecular charge transfer of donor–acceptor-type dye conjugates through GlcNAc modification, fluorogenic probes for OGA have also been developed.^14–17^ However, molecular probes incorporating GlcNAc as the natural substrate generally lack selectivity for OGA over hexosaminidases (HEX), which unselectively hydrolyze hexosamines.^18,19^ This limits their use for accurately measuring OGA activity in complicated biological systems.

Previous research has indicated that increasing the steric hindrance of substituents on the 2-amino group of GlcNAc improves substrate affinity for OGA.^20^ In an earlier study, we have also shown that the replacement of the acetyl (Ac) group in GlcNAc with a propionyl (Pr) group results in a significantly improved selectivity for OGA over HEX in solution-based assays. However, whether this replacement strategy can work for the selective detection of endogenous OGA over HEX in live cells remains unexplored.

**Fig. 1:**
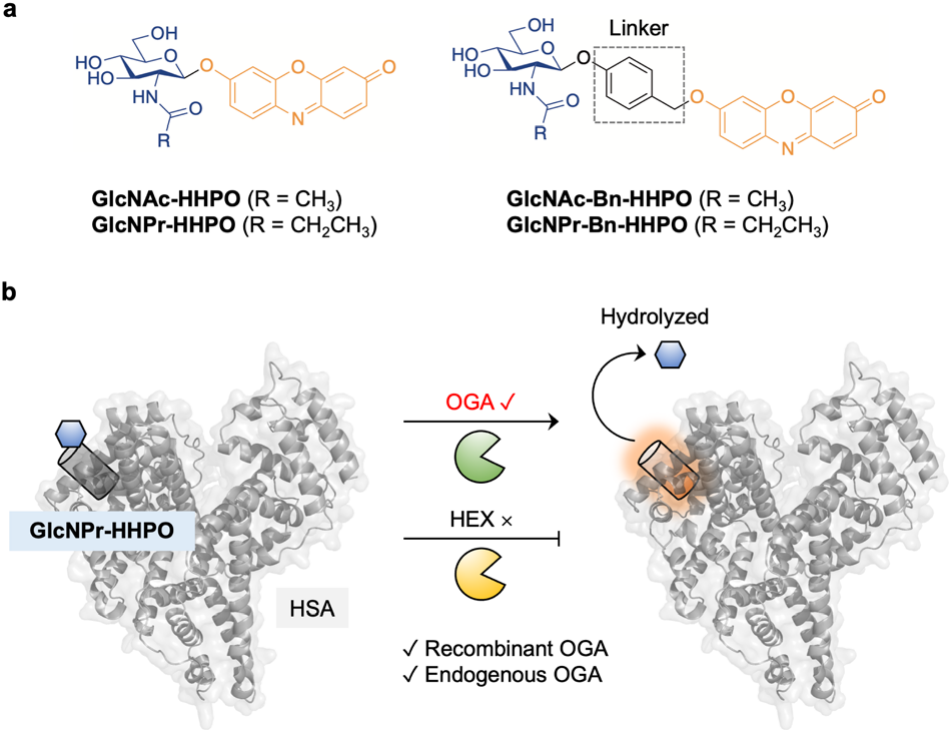
(a) Structure of GlcNAc-HHPO, GlcNPr-HHPO, GlcNAc-Bn-HHPO and GlcNPr-Bn-HHPO. (b) Sensing mechanism of the HSA/GlcNPr-HHPO complex for O-GlcNAcase (OGA) over hexosaminidases (HEX).

Here, we developed a series of fluorogenic probes (GlcNAc-HHPO, GlcNPr-HHPO, GlcNAc-Bn-HHPO and GlcNPr-Bn-HHPO) for the detection of OGA activity in cell lysates and live cells, among which GlcNPr-HHPO with a Pr substitution exhibited outstanding selectivity for OGA over HEX (Fig. 1a). In addition, to improve the sensing performance, our previously developed protein-encapsulation strategy was exploited.^21–23^ Assemblies of our synthesized fluorogenic probes and human serum albumin (HSA) were prepared through host–guest assembly (Fig. 1b). Small-angle X-ray scattering (SAXS) was used to resolve the complex structure between GlcNPr-HHPO and HSA. Importantly, this ensemble achieved the selective detection of endogenous OGA over HEX activity in two cell lines with the results agreeing with that obtained using quantitative real-time polymerase chain reaction (qPCR) analysis. Furthermore, it could accurately sense OGA activity change in live cells.

## Results and discussion

The synthesis of the fluorogenic probes is shown in Scheme S1. Resorufin, which is commonly used for fluorogenic assays, was used as the fluorescent reporter; the introduction of functional groups to its phenolic alcohol quenches the fluorescence due to masking of intramolecular charge transfer.^24–26^ Two series of fluorogenic probes were constructed; one through the direct glycosylation of resorufin with GlcNAc/GlcNPr (GlcNAc-HHPO/GlcNPr-HHPO), and the other exploiting a benzyl (Bn) linker (GlcNAc-Bn-HHPO/GlcNPr-Bn-HHPO). The introduction of the linker aimed to improve sensitivity,^27^ while the replacement of Ac with Pr should lead to selectivity for OGA over HEX. To develop the first series of probes, 1-α-Cl-substituted GlcNAc^17^ and GlcNPr^17^ were treated with resorufin in the presence of Cs_2_CO_3_ and Na_2_SO_4_ in MeCN,^27^ and a subsequent deacetylation using MeONa/MeOH afforded the desired GlcNAc-HHPO and GlcNPr-HHPO, respectively. The second series was similarly constructed by introducing 1-β-O-benzyl-substituted GlcNAc^27^ and GlcNPr^27^ to the phenolic alcohol of resorufin, and subsequent deacetylation gave probes GlcNAc-Bn-HHPO and GlcNPr-Bn-HHPO, respectively.

**Fig. 2:**
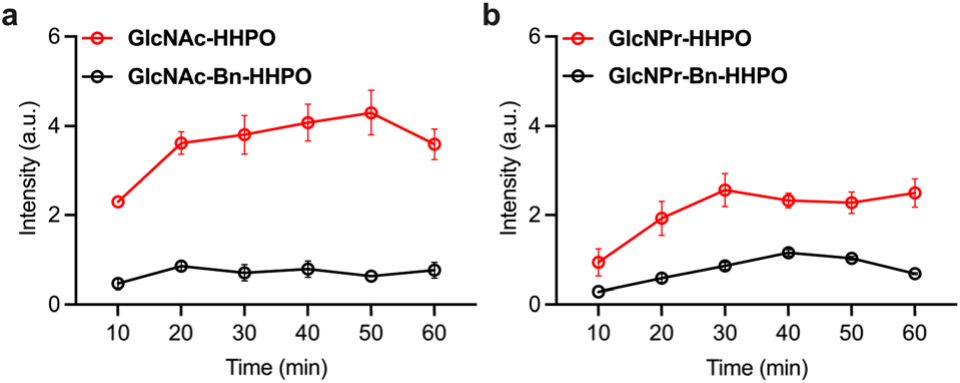
Time-dependent changes in fluorescence emission intensity at 598 nm of (a) GlcNAc-Bn-HHPO (50 µM) and GlcNAc-HHPO (50 µM), and (b) GlcNPr-Bn-HHPO (50 µM) and GlcNPr-HHPO (50 µM) in the presence of OGA (1 µg mL^−1^) in a working buffer (0.1% bovine serum albumin (BSA), 50 mM NaH_2_PO_4_, 100 mM NaCl, pH 7.0) with an excitation wavelength of 550 nm measured by a microplate reader.

With these probes in hand, we established a fluorogenic assay using a purified recombinant human OGA.^18,27^ The probes (50 µM) were incubated with OGA (1 µg mL^−1^) in a working buffer (0.1% bovine serum albumin (BSA), 50 mM NaH_2_PO_4_, 100 mM NaCl, pH 7.0),^18^ and the fluorescence of the resulting mixtures was measured using a microplate reader. According to previous studies, the presence of the Bn linker between the glycosyl substrate and the fluorophore could significantly enhance the sensitivity of the resulting probe for the target glycosidase.^28,29^ However, our analytical results indicated that the fluorescence enhancement of both GlcNAc-HHPO (Fig. 2a) and GlcNPr-HHPO (Fig. 2b) with OGA was much more evident than for GlcNAc-Bn-HHPO and GlcNPr-Bn-HHPO over time, respectively. We attributed this observation to the insufficient solubility of the Bn-containing probes in aqueous solutions. To verify our hypothesis, we obtained the absorption spectra of GlcNPr-HHPO and GlcNPr-Bn-HHPO in phosphate buffered saline (PBS, 0.01 M, pH 7.4) (Fig. S1). Over a concentration range of 10–100 µM, we observed that the absorption of GlcNPr-HHPO enhanced linearly, whereas that of GlcNPr-Bn-HHPO varied slightly. In addition, reddish, insoluble species were generated in a solution of 50 µM of GlcNPr-Bn-HHPO. These results suggest that the suboptimal sensitivity of the probes containing the Bn spacer is probably due to their inferior aqueous solubility, which subsequently compromised enzymatic reactivity.

**Fig. 3:**
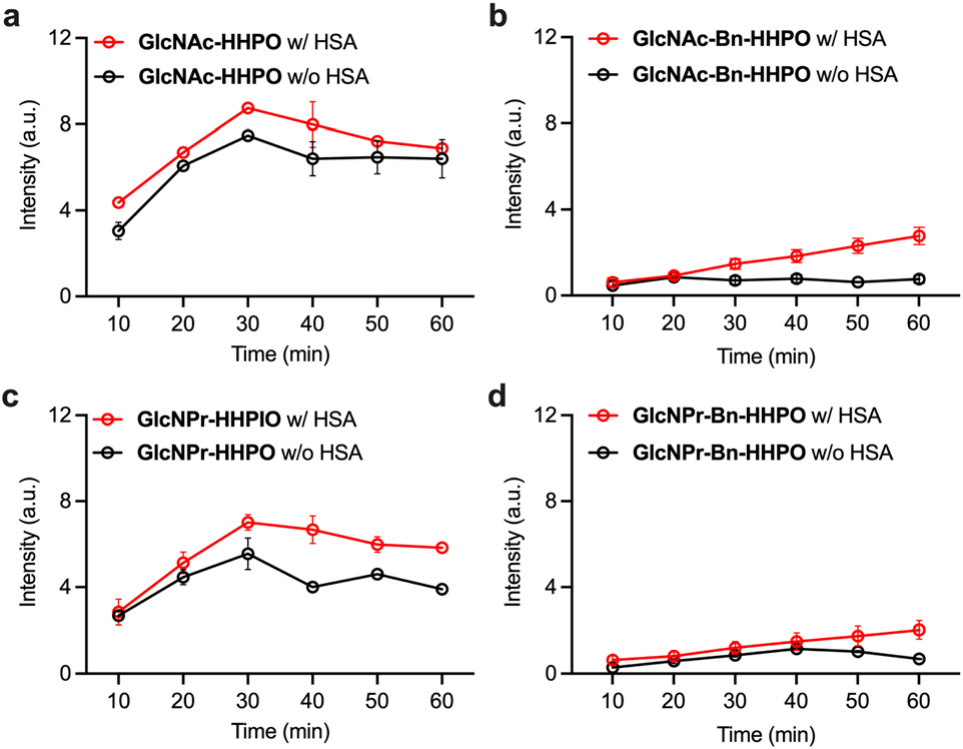
Time-dependent changes in fluorescence emission changes at 598 nm of (a) GlcNAc-Bn-HHPO (50 µM), (b) GlcNAc-HHPO (50 µM), (c) GlcNPr-Bn-HHPO (50 µM) and (d) GlcNPr-HHPO (50 µM) with and without HSA (50 µM) in the presence of OGA (1 µg mL^−1^) in a working buffer (0.1% BSA, 50 mM NaH_2_PO_4_, 100 mM NaCl, pH 7.0) with an excitation wavelength of 550 nm measured by a microplate reader.

Next, we sought to improve the sensitivity of the probes through our previously developed protein-encapsulation strategy.^21–23,30^ HSA, which is among the most abundant proteins found in the human body, was used to encapsulate the probes. The probes were incubated with the albumin in the working buffer with mild stirring for 5 min, producing the ensembles HSA/GlcNAc-HHPO, HSA/GlcNAc-Bn-HHPO, HSA/GlcNPr-HHPO and HSA/GlcNPr-Bn-HHPO. Through fluorescence-based measurements, we determined that the presence of HSA consistently improved the sensitivity of all four probes for OGA over time (Fig. 3). The fact that the addition of HSA improved the sensitivity as well as detection linearity of HSA/GlcNAc-Bn-HHPO (Fig. 3c) and HSA/GlcNPr-Bn-HHPO (Fig. 3d) might be a result of an improved dispersibility of these Bn-containing probes in aqueous buffer solutions. However, the probes without the Bn spacer still exhibited a better sensitivity for OGA. In addition, we chose GlcNPr-HHPO to optimize the mixing ratio with HSA. The results indicated that a HSA:probe ratio of 1 : 1 was optimal for OGA detection (Fig. S2). Subsequently, we used the more sensitive HSA/GlcNAc-HHPO and HSA/GlcNPr-HHPO for selectivity tests. We determined that HSA/GlcNAc-HHPO exhibited a similar level of fluorescence recovery in the presence of OGA and HEX (Fig. S3a). In contrast, while the fluorescence of HSA/GlcNPr-HHPO was observed to significantly enhance when OGA is added, the probe remained quenched with HEX (Fig. S3b). This agrees with our previous study that the replacement of Ac with Pr in GlcNAc substantially improves its selectivity for OGA over HEX.^18^

**Fig. 4:**
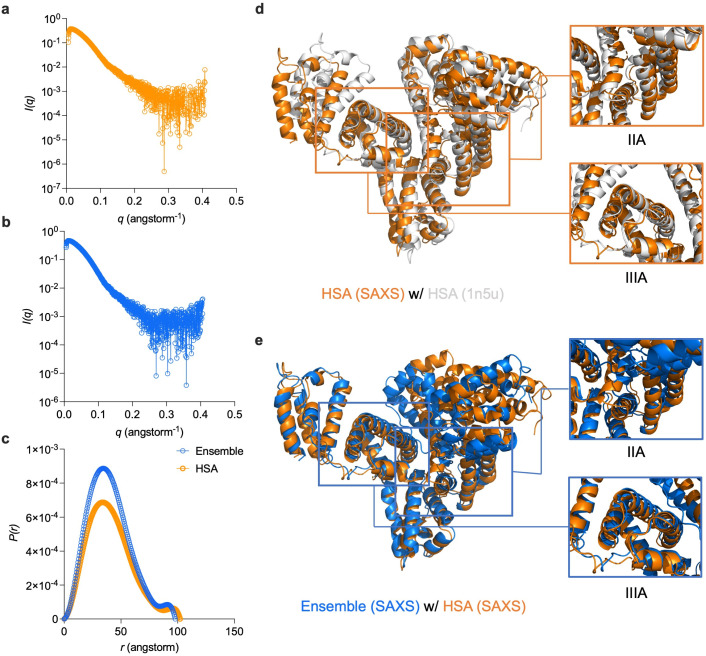
X-ray scattering pattern of (a) HSA, and (b) HSA/GlcNPr-HHPO. (c) Interatomic distance distribution function, *P*(r), of the X-ray scattering patterns of HSA and HSA/GlcNPr-HHPO. (d) Superimposed crystalline structure of HSA (PDB ID: 1n5u) and a simulated atomic model of HSA. (e) Superimposed simulated models of HSA and the HSA/GlcNPr-HHPO ensemble.

In order to gain deeper understanding of the binding mode between GlcNPr-HHPO and HSA, small-angel X-ray scattering (SAXS) was used.^23,28^ The SAXS profile for HSA and that for the HSA/GlcNPr-HHPO ensemble is shown in Fig. 4a and b, respectively. SREFLEX,^31^ a hybrid modelling program that systematically incorporates SAXS data with normal mode analysis was used to simulate the three-dimensional models of HSA and the ensemble. The HSA model was first superimposed onto a reported HSA crystalline structure (PDB entry: 1n5u), and a good overlap between the two models was found (Fig. 4d). Then, the SAXS model of HSA and that of HSA/GlcNPr-HHPO was superimposed to analyze the conformational changes of the latter. As shown in Fig. 4e, while the IIIA region of both models overlapped well, the IIA region of the ensemble was found to position differently from that of HSA. This suggests an inclusion of the probe into the IIA region of HSA, which is known to accommodate hydrophobic molecules.^32^ We also stacked the distance distribution function (*P*(r)) curves of both models (Fig. 4c), and found a decreased maximum diameter (*D*_max_) for HSA/GlcNPr-HHPO (97.9 Å) with respect to HSA (102.1 Å). To corroborate the binding, a competition assay was performed using phenylbutazone (a known IIA-region binder) and ibuprofen (a known IIIA-region binder) as competing agents. The addition of increasing concentrations of phenylbutazone led to the gradual decrease of the tryptophan fluorescence in the IIA site of HSA (Fig. S4),^33^ which correlated with the probe and was taken as evidence for the inclusion of the probe into the IIA region of HSA. The binding constant (*K*_a_) of probe GlcNPr-HHPO with HSA was determined to be 3.685 × 10^5^ M^−1^ using the fluorescence double reciprocal method (Fig. S5).

**Fig. 5:**
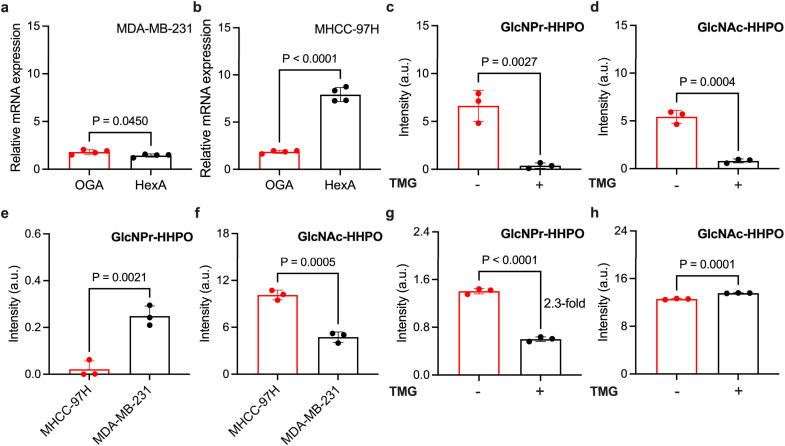
Relative mRNA level of OGA and HexA in (a) MDA-MB-231, and (b) MHCC-97H cells determined by qPCR. Measuring the fluorescence intensity at 598 nm of (e) HSA/GlcNPr-HHPO (50 µM/50 µM), and (f) HSA/GlcNAc-HHPO (50 µM/50 µM) in MDA-MB-231 and MHCC-97H cell lysates in a working buffer (0.1% BSA, 50 mM NaH_2_PO_4_, 100 mM NaCl, pH 7.0) with an excitation wavelength of 598 nm. Fluorescence intensity at 598 nm of (c) HSA/GlcNPr-HHPO (50 µM/50 µM) and (d) HSA/GlcNAc-HHPO (50 µM/50 µM) incubated with OGA (1 µg ml^−1^) in the absence and presence of TMG (21 nM) for 1 h. Fluorescence intensity at 598 nm of (g) HSA/GlcNPr-HHPO (50 µM/50 µM) and (h) HSA/GlcNAc-HHPO (50 µM/50 µM) incubated in the cell lysate of MDA-MB-231 in the absence and presence of TMG (21 nM) for 1 h. Statistical analysis was performed with two-sided Student's *t*-test.

With the promising sensing properties of the ensembles determined, we turned our attention to evaluate their ability to measure endogenous OGA activity in cells. Two cell lines, MDA-MB-231 (human triple-negative breast cancer) and MHCC-97H (human liver cancer), with different OGA and HEX expression levels were used. We first used qPCR to determine the endogenous expression level of OGA and HexA (a predominant isozyme of HEX) in the two cell lines. The results indicated a slightly higher expression level of OGA than that of HexA in MDA-MB-231 (Fig. 5a), and a ∼4-fold higher HexA level than OGA in MHCC-97H (Fig. 5b).

Next, we used both HSA/GlcNAc-HHPO and HSA/GlcNPr-HHPO to treat the cell lysates. Interestingly, we determined a ∼8-fold higher fluorescence for HSA/GlcNPr-HHPO in MDA-MB-231 than MHCC-97H (Fig. 5e). In contrast, a ∼2-fold stronger fluorescence was seen for HSA/GlcNAc-HHPO in MHCC-97H than MDA-MB-231 (Fig. 5f). These results suggest that (1) the fluorescence activation of HSA/GlcNAc-HHPO is largely dependent on the HEX expression of the cells because HexA was determined to be predominantly expressed in MHCC-97H, and (2) HSA/GlcNPr-HHPO can be used to more accurately measure endogenous OGA activity irrespective of HEX expression as evidenced by its minimal fluorescence recovery in the lysate of MHCC-97H cells.

To better corroborate the OGA selectivity of the ensembles, a selective OGA inhibitor, Thiamet-G (TMG) with a nanomolar half-maximal inhibitory concentration, was used.^34^ We first found that TMG inhibited the fluorescence enhancement of both HSA/GlcNAc-HHPO (Fig. 5d) and HSA/GlcNPr-HHPO (Fig. 5c) when incubated with recombinant OGA. This confirms the effectiveness of the inhibitor. Then, the cell lysate of MDA-MB-231 was collected and pre-treated with TMG, followed by incubation with the ensembles. While treatment of TMG did not interrupt the fluorescence recovery of HSA/GlcNAc-HHPO (Fig. 5h), a ∼2.3-fold fluorescence decrease was determined for HSA/GlcNPr-HHPO in the presence of TMG (Fig. 5g). This demonstrates that HSA/GlcNPr-HHPO but not HSA/GlcNAc-HHPO could selectively detect OGA activity irrespective of the expression of HEX.

**Fig. 6:**
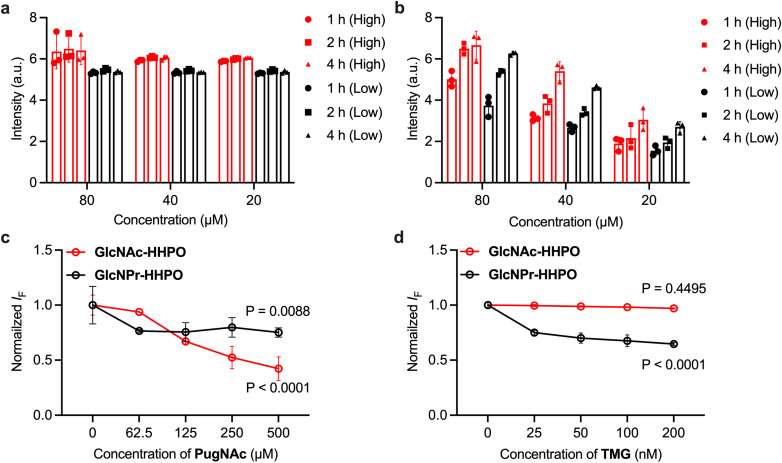
Incubation of HeLa cells with (a) HSA/GlcNAc-HHPO and (b) HSA/GlcNPr-HHPO at different concentrations with high (80–90% per well) and low (40–50% per well) cell densities for 1,2 and 4 h. Normalized fluorescence intensity of HeLa cells incubated with HSA/GlcNAc-HHPO (50 µM/50 µM) and HSA/GlcNPr-HHPO (50 µM/50 µM) in the presence of (c) PugNAc and (d) TMG with different concentrations for 2 h. All measurements were done in a microplate reader with an excitation wavelength of 550 nm (the fluorescence emission intensity at 598 nm was measured). Statistical analysis was performed using two-way ANOVA and Dunnett's multiple comparisons test (*n* = 3; the *P* values shown in the diagrams were obtained with respect to the group in which the inhibitor was absent).

To further evaluate whether our probes could accurately report on OGA activities in live cells, the following experiments were carried out (results are shown in Fig. 6). HeLa (human cervix) cells with a higher endogenous expression level of HexA than that of OGA were used (Fig. S6). The cells were incubated at different densities (40–50% or 80–90% per well) with both HSA/GlcNAc-HHPO (Fig. 6a) and HSA/GlcNPr-HHPO (Fig. 6b). We found that in all test groups the fluorescence of HSA/GlcNAc-HHPO reached equilibrium in 1 h irrespective of cell density and probe concentration. In contrast, the fluorescence enhancement of HSA/GlcNPr-HHPO was seen to be dependent on both the concentration of the probe and cell density. This suggests a quicker intracellular hydrolysis of HSA/GlcNAc-HHPO than that of HSA/GlcNPr-HHPO probably due to the action of both OGA and HEX. A cell viability assay showed that the probes were not toxic to HeLa cells over a concentration range of 0–80 µM (Fig. S7). In addition, a competition assay was carried out by pre-treating HeLa cells with two known glycosidase inhibitors – PugNAc that unselectively inhibits OGA and HEX, and TMG that selectively inhibits OGA.^34^ Interestingly, the results shown in Fig. S6c (PugNAc) and Fig. S6d (TMG) clearly indicate that the fluorescence of both probes decreased with increasing concentrations of PugNAc; however, in the presence of the OGA selective inhibitor, TMG, only the fluorescence of HSA/GlcNPr-HHPO was seen to decrease in a concentration-dependent manner.

## Conclusions

We have developed resorufin-based fluorogenic probes for OGA. We found that the presence of a benzyl spacer compromised the sensitivity of the probes probably due to solubility issues. However, association with HSA was effective in improving the sensitivity of the probes for OGA. Importantly, the substitution of Pr for the Ac group gave rise to selectivity for OGA over HEX. This enables us to differentiate between two cell lines with different endogenous OGA and HexA expression levels. While, comparing with previously established OGA probes (Table S1), the probes also achieved the selective detection of OGA in live cells. As such our research provides valuable chemical tools for the determination of OGA activity in complicated biological systems, and offers insight into the development of molecular glycoprobes for other discrete glycosidases.^35,36^

## Author contributions

Yuan-Hao Wu, Guo Chen and Zi-Ru Ye performed experiments, analyzed data and drafted the manuscript. Xi-Le Hu, Tony D. James, Jia Li and Xiao-Peng He supervised research and edited the manuscript. All authors have approved the final version of the manuscript.

## Conflicts of interest

There are no conflicts to declare.

## Supplementary Material

SC-OLF-D5SC06843F-s001

## Data Availability

All data supporting this research are available as part of the supplementary information (SI). Supplementary information: experimental methods, additional figures, and spectra of new compounds. See DOI: https://doi.org/10.1039/d5sc06843f.
