# Supplementary material for: Protein-encapsulated fluorogenic probes for the selective detection of endogenous O-GlcNAcase (OGA)
Source: Chem Sci. 2026 Feb 17;17(14):7178–84. doi: 10.1039/d5sc06843f (PMC12910607; doi:10.1039/d5sc06843f)
Supplement: SC-017-D5SC06843F-s001 [file SC-017-D5SC06843F-s001.pdf]

Supplementary Information (SI) for Chemical Science.
This journal is © The Royal Society of Chemistry 2026

Supporting Information

Protein-encapsulated fluorogenic probes for the selective detection of endogenous O-GlcNAcase (OGA)

Yuan-Hao Wu,[1] Chen Guo,[1,3] Zi-Ru Ye,[1,3] Xi-Le Hu,[1] Tony D James,[4,5,*] Jia Li[3,*] and Xiao-Peng He[1,2,*]

[1] *Key Laboratory for Advanced Materials and Joint International Research Laboratory of Precision Chemistry and Molecular Engineering, Feringa Nobel Prize Scientist Joint Research Center, School of Chemistry and Molecular Engineering, East China University of Science and Technology, 130 Meilong Rd, Shanghai 200237, China*

[2] *The International Cooperation Laboratory on Signal Transduction, Eastern Hepatobiliary Surgery Hospital, National Center for Liver Cancer, Shanghai 200438, China*

[3] *National Center for Drug Screening, State Key Laboratory of Drug Research, Shanghai Institute of Materia Medica, Chinese Academy of Sciences, Shanghai 201203, China*

[4] *Deparment of Chemistry, University of Bath, Bath, BA2 7AY, U.K.*

[5] *School of Chemistry and Chemical Engineering, Henan Normal University, Xinxiang 453007, China*

[a] *Equal contribution*

Email addresses

t.d.james@bath.ac.uk (T. D. James)

jli@simm.ac.cn (J. Li)

xphe@ecust.edu.cn (X.-P. He)

<u>Content list</u>

S1. Experimental section
S2. Additional figures
S3. Original spectra of new compounds
S4. Additional References

S1. Experimental section

General. All chemicals and reagents were purchased commercially at analytical grade unless otherwise noted. Human serum albumin (HSA) and *N*-acetyl-β-D-hexosaminidase (from *Canavalia ensiformis*) were purchased from Shanghai Yuanye Bio-Technology Company (#S10237). ^{1}H NMR and ^{13}C NMR spectra were recorded on a Bruker AM 400MHz spectrometer with tetramethylsilane (TMS) as the internal reference. Fluorogenic assays were done on a M5 microplate reader.

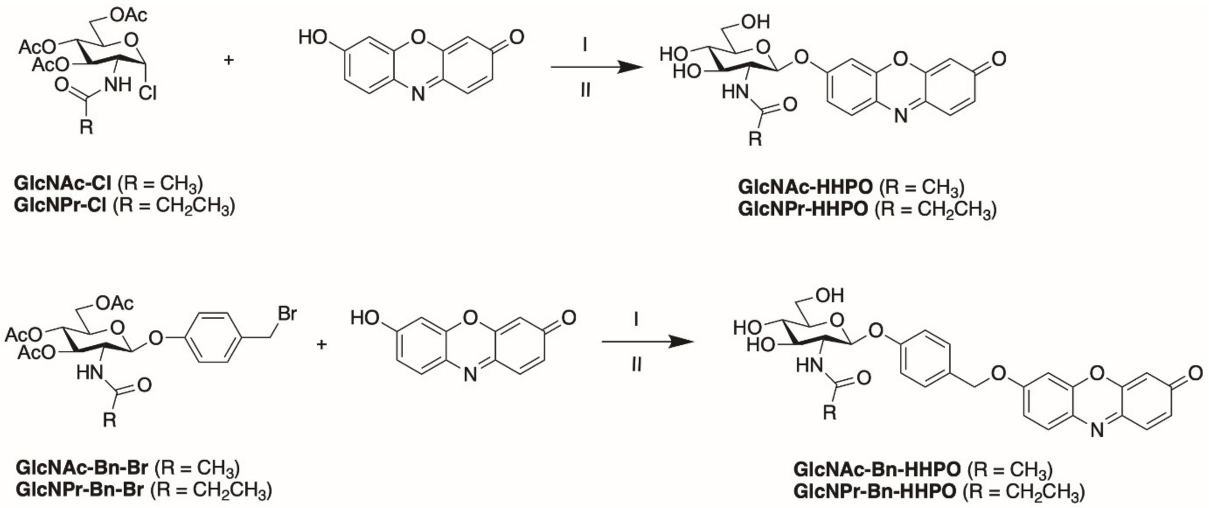

Scheme S1. Synthesis of the OGA probes. Reagents and conditions: (I) Cs_2CO_3, and Na_2SO_4 in MeCN at r.t. (II) MeONa in MeOH.

General procedure for the synthesis of GlcNPr-HHPO, GlcNAc-Bn-HHPO and GlcNPr-Bn-HHPO. A mixture of glycoside (2.0 eq.), **HHPO** (1.0 eq.), Cs_2CO_3 (5.0 eq.) and Na_2SO_4 (4.0 eq.) in MeCN was vigorously stirred overnight. Then, solvent was removed under reduced pressure. The resulting residue was re-dissolved in DCM (30 mL), and washed with water (30 mL) and then brine (30 mL). The organic layer was collected, dried with Na_2SO_4 and concentrated in vacuum to obtain a crude product. The crude product was purified by column

chromatography (DCM/MeOH = 100/1 to 95/5, v/v). The resulting product was then dissolved in MeOH (5 mL), followed by addition of MeONa (3 equiv.) at 0 °C. The reaction mixture was stirred at room temperature for 2 h, and then recrystallized in DCM (10 mL) and then MeOH (10 mL) to afford the final product.

Synthesis of GlcNAc-HHPO. The known compound **GlcNAc-HHPO** was synthesized according to a previous report.[2]

Synthesis of GlcNPr-HHPO. GlcNPr-HHPO was obtained as a deep-brown solid (28.0 mg, 46% yield over two steps) from **GlcNPr-Cl** (100 mg, 0.26 mmol), **HHPO** (20 mg, 0.09 mmol), Cs_2CO_3 (158 mg, 0.4 mmol) and Na_2SO_4 (55 mg, 0.36 mmol) according to the general procedure above. ^{1}H NMR (400 MHz, DMSO-d_6) δ 7.77 (d, J = 8.9 Hz, 1H), 7.74 (d, J = 9.0 Hz, 1H), 7.53 (d, J = 9.8 Hz, 1H), 7.08 (d, J = 2.5 Hz, 1H), 7.00 (dd, J = 8.9, 2.6 Hz, 1H), 6.79 (dd, J = 9.8, 2.1 Hz, 1H), 6.27 (d, J = 2.0 Hz, 1H), 5.20–5.13 (m, 3H), 5.11 (d, J = 5.6 Hz, 1H), 4.66 (t, J = 5.7 Hz, 1H), 3.78–3.66 (m, 2H), 3.52–3.47 (m, 1H), 3.47–3.39 (m, 1H), 3.22–3.17 (m, 1H), 2.05 (q, J = 7.5 Hz, 2H), 0.96 (t, J = 7.6 Hz, 3H); ^{13}C NMR (101 MHz, DMSO-d_6) δ 185.9, 173.7, 161.4, 150.1, 146.4, 145.4, 135.5, 134.4, 131.8, 129.1, 115.3, 106.2, 103.1, 99.4, 77.9, 74.2, 70.6, 61.1, 55.6, 29.4, 10.5. HRMS (ESI, *m/z*): $[M + Na]^+$: calculated for $C_{21}H_{22}N_2O_8Na^+$ 453.1274, found 453.1271.

Synthesis of GlcNAc-Bn-HHPO. GlcNAc-Bn-HHPO was obtained as a deep-brown solid (24.0 mg, 51% yield over two steps) from **GlcNAc-Bn-Br** (100 mg, 0.19 mmol), **HHPO** (20 mg, 0.09 mmol), Cs_2CO_3 (158 mg, 0.4 mmol) and Na_2SO_4 (55 mg, 0.36 mmol) according to the general procedure above. ^{1}H NMR (400 MHz, DMSO-d_6) δ 7.81 (d, J = 9.0 Hz, 1H), 7.75 (d, J = 8.9 Hz, 1H), 7.51 (d, J = 9.8 Hz, 1H), 7.44–7.37 (m, 2H), 7.16 (d, J = 2.6 Hz, 1H), 7.09 (dd, J = 8.9, 2.6 Hz, 1H), 6.98 (d, J = 8.6 Hz, 2H), 6.77 (dd, J = 9.8, 2.1 Hz, 1H), 5.26–5.04 (m, 2H), 4.97 (d, J = 8.4 Hz, 1H), 4.75–4.51 (brs, 1H), 3.75–3.59 (m, 1H), 3.51–3.44 (m, 1H), 3.43–3.19 (m, 1H), 3.32–3.26 (m, 1H), 3.17 (t, J = 9.2 Hz, 1H), 1.79 (s, 3H); ^{13}C NMR (101 MHz, DMSO) δ 185.8, 169.7, 158.0, 150.2, 145.7, 135.4, 134.2, 131.8, 130.2, 129.9, 116.9, 114.8, 106.1, 101.6, 99.5, 77.7, 74.5, 70.7, 70.5, 61.2, 56.8, 55.9, 23.6. HRMS (ESI, *m/z*): $[M]^-$: calculated for $C_{27}H_{25}N_2O_9$ 521.1560, found 521.1558.

Synthesis of GlcNPr-Bn-HHPO. GlcNPr-Bn-HHPO was obtained as a deep-brown solid (25.7 mg, 53% yield over two steps) from **GlcNPr-Bn-Br** (100 mg, 0.19 mmol), **HHPO** (20 mg, 0.09 mmol), Cs_2CO_3 (158 mg, 0.4 mmol) and Na_2SO_4 (55 mg, 0.36 mmol) according to

the general procedure above. ^{1}H NMR (400 MHz, DMSO-d_6) δ 7.79–7.67 (m, 2H), 7.50 (d, J = 9.8 Hz, 1H), 7.40 (d, J = 8.2 Hz, 2H), 7.15 (s, 1H), 7.08 (d, J = 8.7 Hz, 1H), 6.97 (d, J = 8.2 Hz, 2H), 6.76 (d, J = 9.8 Hz, 1H), 5.25–5.02 (m, 2H), 4.96 (d, J = 8.4 Hz, 1H), 4.75–4.51 (brs, 1H), 3.75–3.62 (m, 2H), 3.48–3.41 (m, 1H), 3.52–3.42 (m, 1H), 3.32–3.25 (m, 1H), 3.17 (t, J = 9.0 Hz, 1H), 2.05 (q, J = 7.5 Hz, 2H), 0.97 (t, J = 7.6 Hz, 3H); ^{13}C NMR (101 MHz, DMSO-d_6) δ 185.8, 173.5, 158.1, 150.2, 145.6, 135.4, 134.2, 131.8, 130.2, 129.9, 116.9, 114.8, 106.1, 101.6, 99.9, 77.8, 74.4, 70.8, 70.5, 61.2, 56.9, 55.8, 29.4, 10.5. HRMS (ESI, *m/z*): $[M]^-$: calculated for $C_{28}H_{27}N_2O_9$ 535.1717, found 535.1711.

Preparation of HSA/probe ensembles. Stock solution of probes (10 mM) was prepared in DMSO. Stock solution of HSA (2 mM) was prepared in deionized water. Briefly, to prepare the ensembles, equimolar probe and HSA solutions were added to a working buffer (0.1% bovine serum albumin (BSA), 50 mM NaH_2PO_4, 100 mM NaCl, pH 7.0), and the mixture was incubated and stirred for 5 min to permit sufficient host-guest inclusion.

OGA expression and purification. Recombinant human OGA used for analysis in this study was expressed and purified according to our previously reported procedure.[1]

Fluorogenic assays. A fluorogenic probe (50 μM) or its ensemble with HSA (50 μM/50 μM) was incubated with OGA (1 μg ml^{-1}) or cell lysates in a 384-well microplate with black edge and opaque bottom (Beyotime Biotechnology). Then, the fluorescence of the resulting mixture was measured on a M5 microplate reader. Data were analyzed by Prism software package (GraphPad Prism 9.5.0).

Cell culture. MDA-MB-231 (human triple-negative breast cancer) cells and HeLa (human cervix) cells were obtained from ATCC, and MHCC-97H (human hepatocellular carcinoma) cells was obtained from National Collection of Authenticated Cell Cultures. All cells were cultured in DMEM-HG medium supplemented with 10% fetal bovine serum and 0.1% Penicillin-Streptomycin Solution at 37 °C in a humidified atmosphere with 5% CO_2 and 95% air. Cells were split every two days.

Determination of the mRNA level of OGA and HexA in different cell lines. Cells were lysed by TRIzol reagent, and then total RNA were isolated by chloroform and isopropanol. Then, reverse transcription afforded the corresponding cDNA. Finally, real-time quantitative polymerase-chain reaction (qPCR) was performed with Genious 2X SYBR Green Fast qPCR Mix (Abclonal Inc.) using a Real-Time PCR system (LightCycler® 96 Instrument, Roche).

The primer sequences used are as follows.

Gene	Forward (5' to 3')	Reverse (5' to 3')
**β-Actin**	ATCACCATTGGCAATGAGCG	TTGAAGGTAGTTTCGTGGAT
**OGA**	GCAAGAGTTTGGTGTGCCTCATC	GTGCTGCAACTAAAGGAGTCCC
**HexA**	GGAGGTCATTGAATACGCACGG	GGATTCACTGGTCCAAAGGTGC

Cell lysates. Cells at a density of 2×10^6 were collected and treated with radio-immunoprecipitation assay (RIPA) lysis buffer supplied with 1 mM phenylmethylsulfonyl fluoride (PMSF). Then, samples were centrifuged at 14000 × g for 15 min at 4 °C to precipitate cell debris. After centrifugation, the supernatant was collected for analysis. The concentration of cell-derived proteins was determined by Coomassie blue staining.

Small-angle X-ray scattering (SAXS). SAXS was performed at beamline BL19U2 of the National Facility for Protein Science Shanghai (NFPS) at the Shanghai Synchrotron Radiation Facility (SSRF). The wavelength, λ, of the X-ray radiation was set at 1.033 Å. Scattered X-ray intensities were collected using a Pilatus 1M detector (DECTRIS Ltd). The sample-to-detector distance was set such that the detecting range of momentum transfer [$q = 4\pi\sin\theta/\lambda$, where 2θ is the scattering angle] of the SAXS experiments was 0.01-0.40 $Å^{-1}$. To reduce the radiation damage, a flow cell made of a cylindrical quartz capillary with a diameter of 1.5 mm and a wall thickness of 10 μm was used. HSA was prepared at 50 μM in phosphate buffered saline (PBS) (10 mM, pH 7.4) in the presence or absence of 50 μM **GlcNPr-HHPO**. The scattering profiles for a matched buffer were subtracted from that of the protein. For each experiment, the SAXS data were collected as 20 × 1 sec exposures. The 2-D scattering images were converted to 1-D SAXS curves through azimuthally averaging after applying a solid angle correction, and then normalizing with the intensity of the transmitted X-ray beam using the software package BioXTAS RAW. Background scattering was subtracted using the PRIMUS program in the ATSAS software package. The conformational change of the hybrid site was observed using the SREFLEX program within the ATSAS software package. Pymol was used to superimpose the three-dimensional SAXS structure of HSA onto a known HSA crystalline structure (PDB ID: 1n5u) as detailed in the main text.

OGA detection in living cells. HeLa cells were seeded on a clear bottom 96-well plate and cultured overnight to achieve different cell densities (40%–50% or 80%–90%). Cells were incubated with different concentrations (0, 20, 40 and 80 μM) of **HSA/GlcNAc-HHPO** or

HSA/GlcNPr-HHPO at 37 °C for 1, 2 and 4 h. Fluorescence images were recorded by a M5 microplate reader. The excitation/emission used were 550/598 nm, respectively.

Inhibition assay. HeLa cells were seeded on a clear bottom 96-well plate and cultured overnight. Cells were incubated with different concentrations of **PugNAc** (0, 62.5, 125, 250 and 500 μM) or **TMG** (0, 25, 50, 100 and 200 nM) for 48 h, and then **HSA/GlcNAc-HHPO** (40 μM/40 μM) or **HSA/GlcNPr-HHPO** (40 μM/40 μM) was added and incubated at 37 °C for another 2 h. Fluorescence images were recorded by a M5 microplate reader. The excitation/emission used were 550/598 nm, respectively.

Cell viability. Cell Counting Kit-8 (CCK-8) assay was performed to test the viability of HeLa cells after treatment with different probes. Cells were seeded onto clear bottom 96-well plates at different densities and cultured overnight. Then, each well was incubated with different concentrations of **HSA/GlcNAc-HHPO** and **HSA/GlcNPr-HHPO** (10, 20, 40 and 80 μM) for 24 hr. Then, 10 μL of CCK-8 reagent was added and incubated for 1 hr. The absorbance of each well was measured at 450 nm using a M5 plate reader. Cell viability is directly proportional to the number of viable cells.

S2. Additional figures

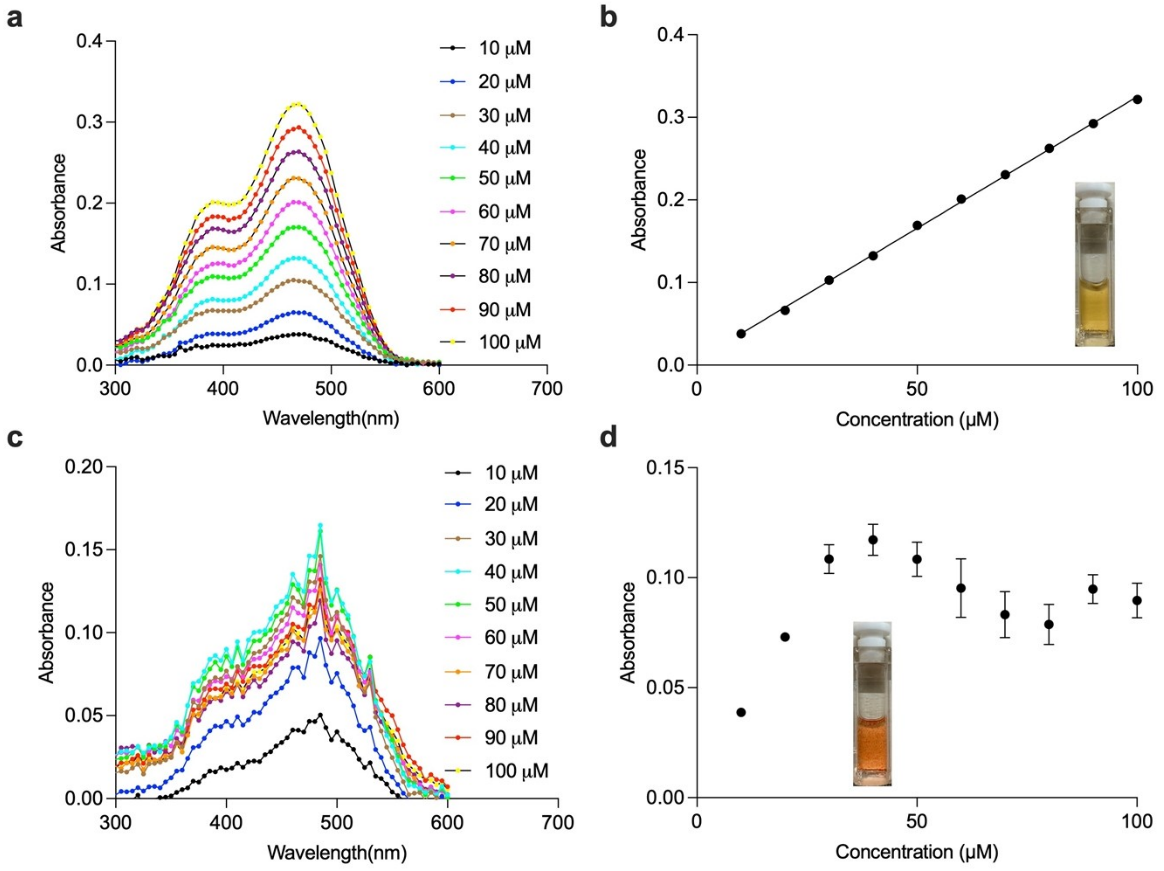

Figure S1. UV-vis absorption spectra of (a) **GlcNPr-HHPO** and (c) **GlcNPr-Bn-HHPO** with increasing concentrations (10 to 100 μM) in PBS (0.01 M, pH 7.4). Plotting the absorbance of (b) **GlcNPr-HHPO** and (d) **GlcNPr-Bn-HHPO** at 470 nm as a function of concentration; the inset photos are the PBS solution of probes (100 μM).

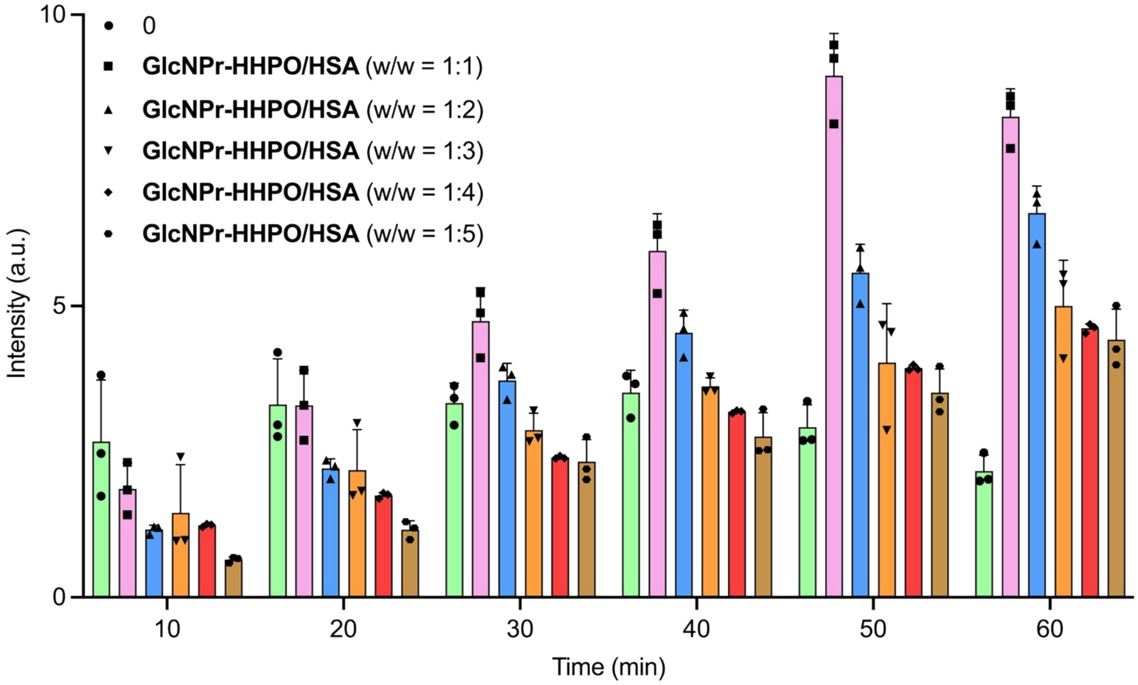

Figure S2. Fluorescence intensity of **GlcNPr-HHPO** (50 μM) at 598 nm without and with HSA at different molar ratios (1:1 to 5:1) after incubation with OGA (1 μg ml^{-1}) for 10–60 min. All measurements were done in a working buffer (0.1% bovine serum albumin (BSA), 50 mM NaH_2PO_4, 100 mM NaCl, pH 7.0) with an excitation wavelength of 550 nm.

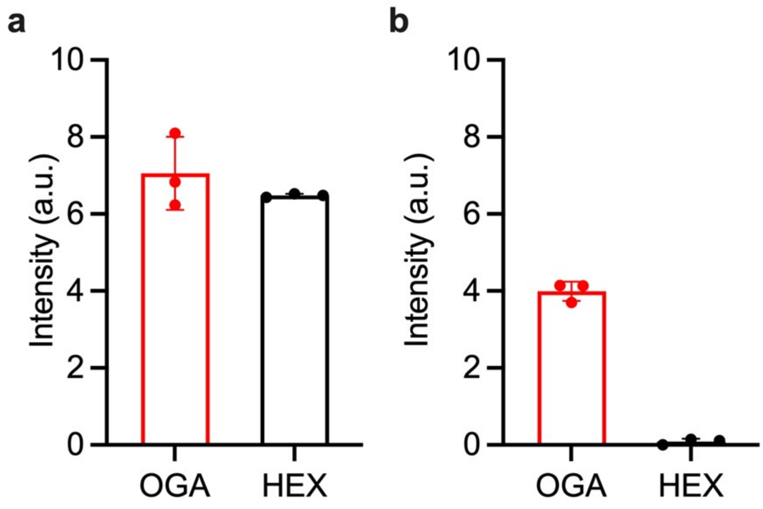

Figure S3. Fluorescence intensity of (a) **HSA/GlcNAc-HHPO** (50 μM/50 μM), and (b) **HSA/GlcNPr-HHPO** (50 μM/50 μM) after incubation with OGA (1 μg ml^{-1}) or HEX (0.03 U $μg^{-1}$) for 1 h. All measurements were done in a working buffer (0.1% BSA, 50 mM NaH_2PO_4, 100 mM NaCl, pH 7.0) with an excitation wavelength of 550 nm.

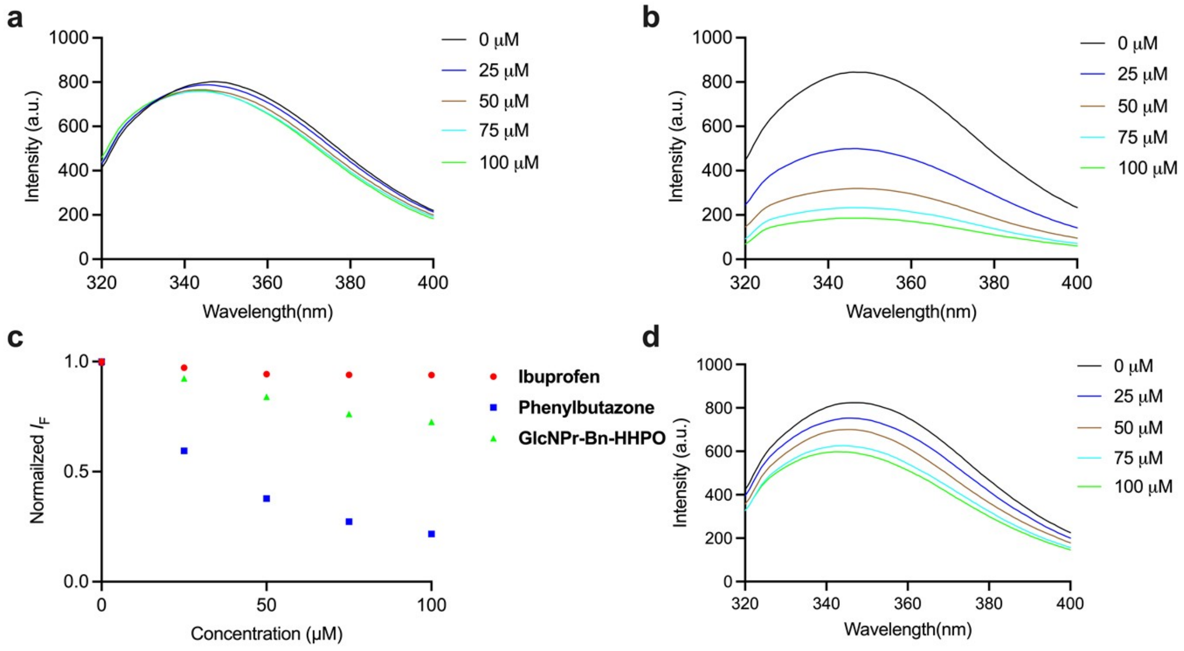

Figure S4. Fluorescence spectra of HSA (50 μM) with increasing (a) ibuprofen (0-100 μM), (b) phenylbutazone (0-100 μM), and (c) **GlcNPr-HHPO** (0-100 μM). All measurements were done in in PBS (0.01 M, pH 7.4) with an excitation wavelength of 295 nm.

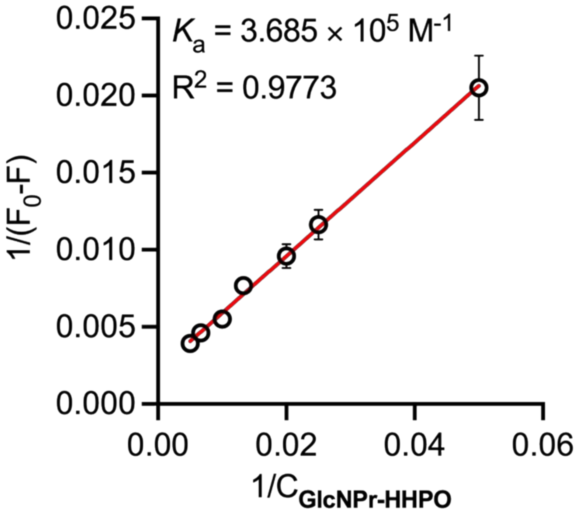

Figure S5. Double reciprocal plot of the fluorescence changes of different concentrations of **GlcNPr-HHPO** in the presence of HSA (50 μM).

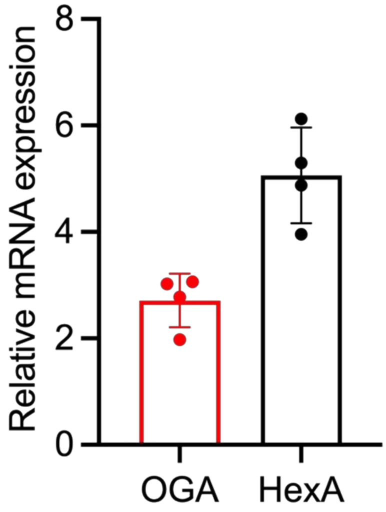

Figure S6. Relative mRNA level of OGA and HexA in HeLa cells.

Structure	Excitation Wavelength (nm)	Emission Wavelength (nm)	Application	Selectivity for OGA over HEX	Reference
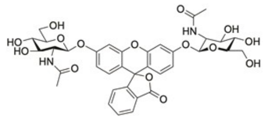	485	535	In vitro	No	2
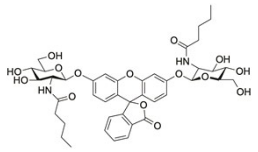	485	535	In vitro	Yes	3
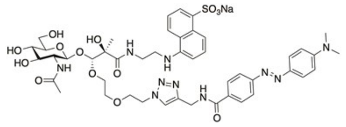	350	490	Cell lysates	No	4
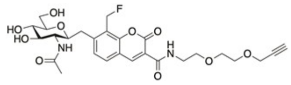	405	450	Living cell	No	5
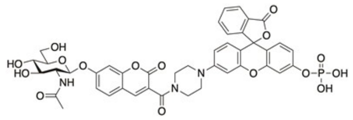	488	520	Living cell	No	6
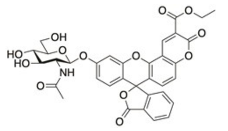	400	450	Living cell	No	7

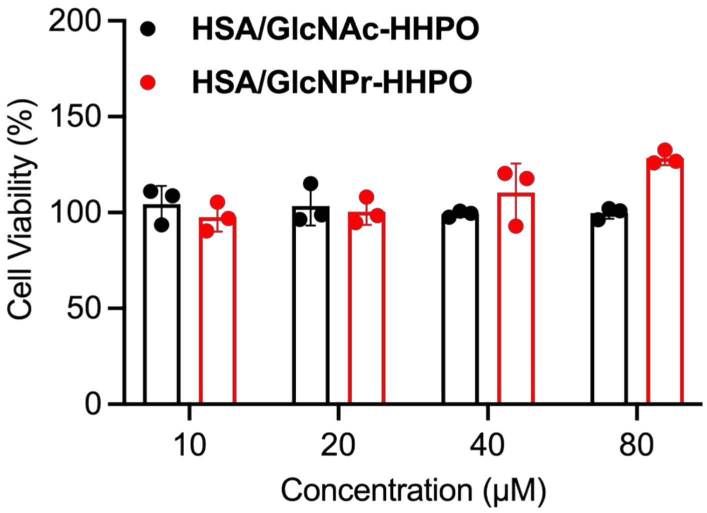

Figure S7. Cell viability of HeLa cells treated with increasing concentrations of **HSA/GlcNPr-HHPO** for 24 h, measured by CCK-8 assay.

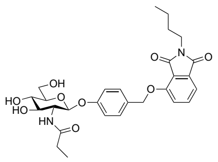	400	515	In vitro	Yes	8
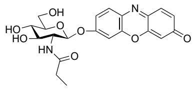	550	598	Cell lysates and living cell	Yes	This work

Table S1. Summary of previously reported OGA probes.

S3. Original spectra of new compounds

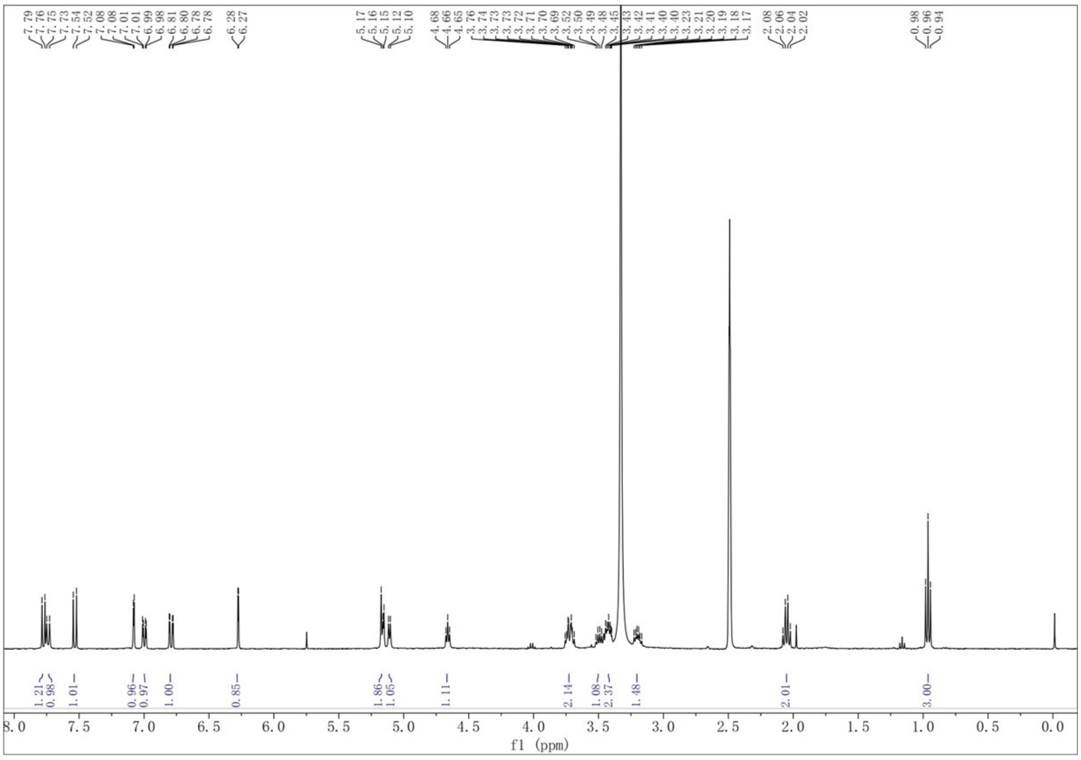

Figure S8. ^{1}H NMR of **GlcNPr-HHPO** in DMSO-d_6.

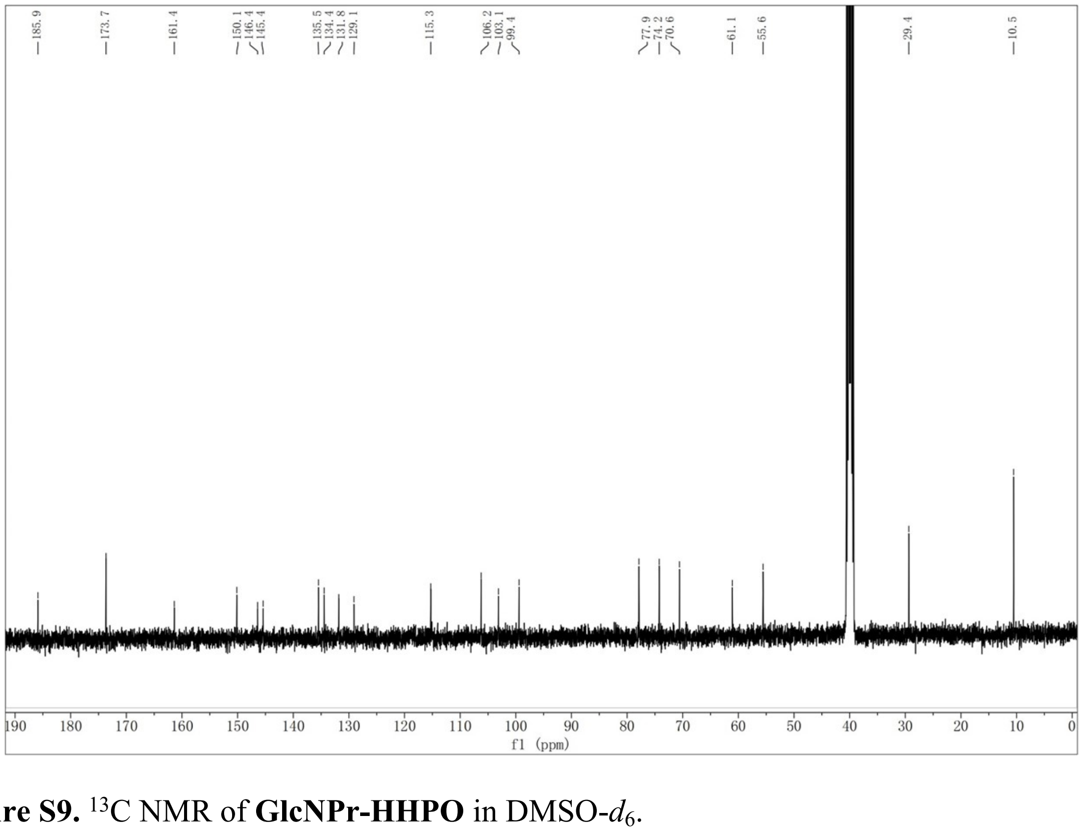

Figure S9. ^{13}C NMR of **GlcNPr-HHPO** in DMSO-d_6.

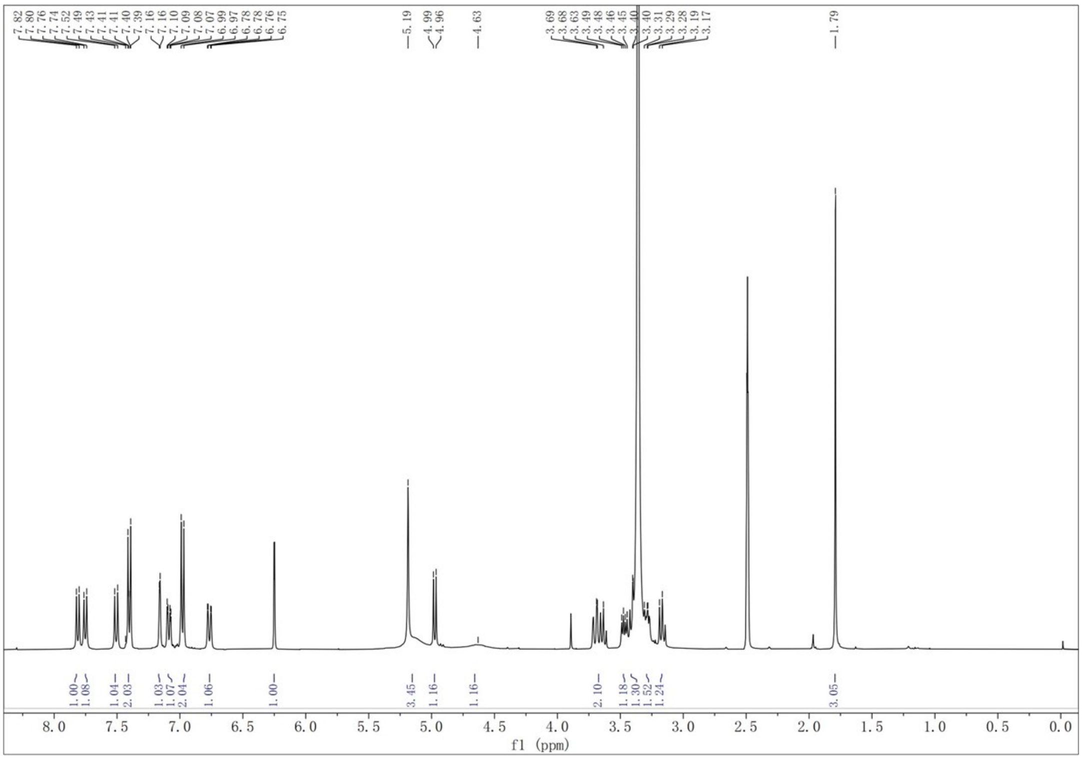

Figure S10. ^{1}H NMR of **GlcNAc-Bn-HHPO** in DMSO-d_6.

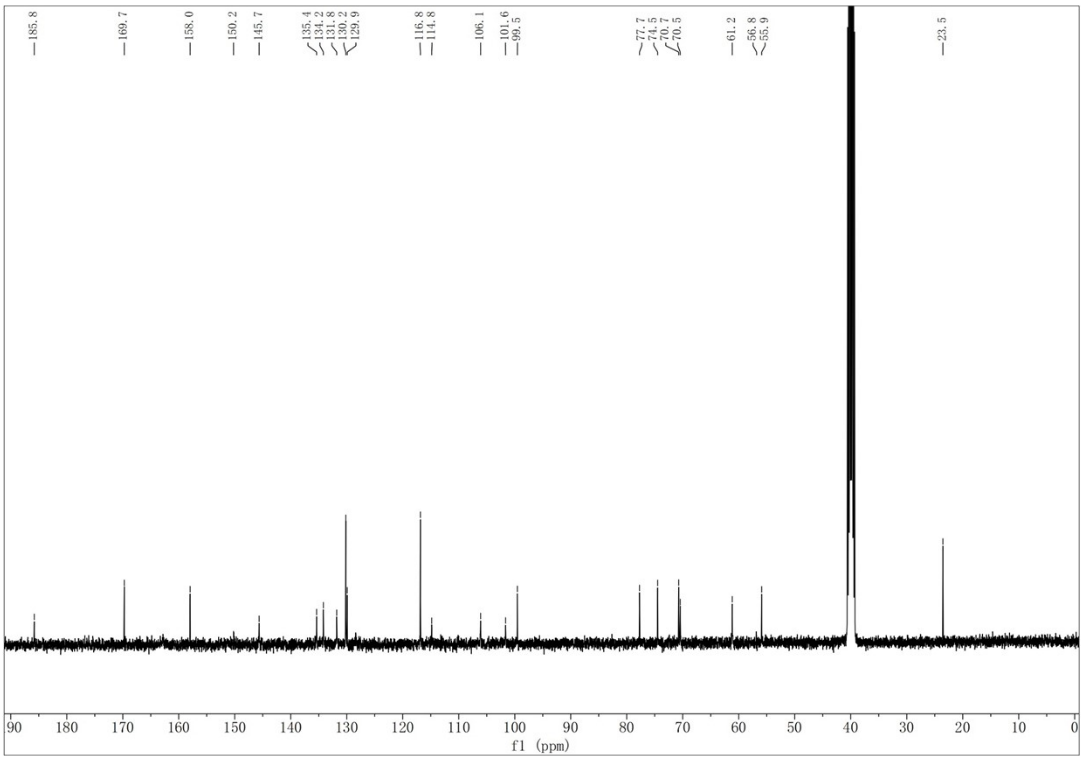

Figure S11. ^{13}C NMR of **GlcNAc-Bn-HHPO** in DMSO-d_6.

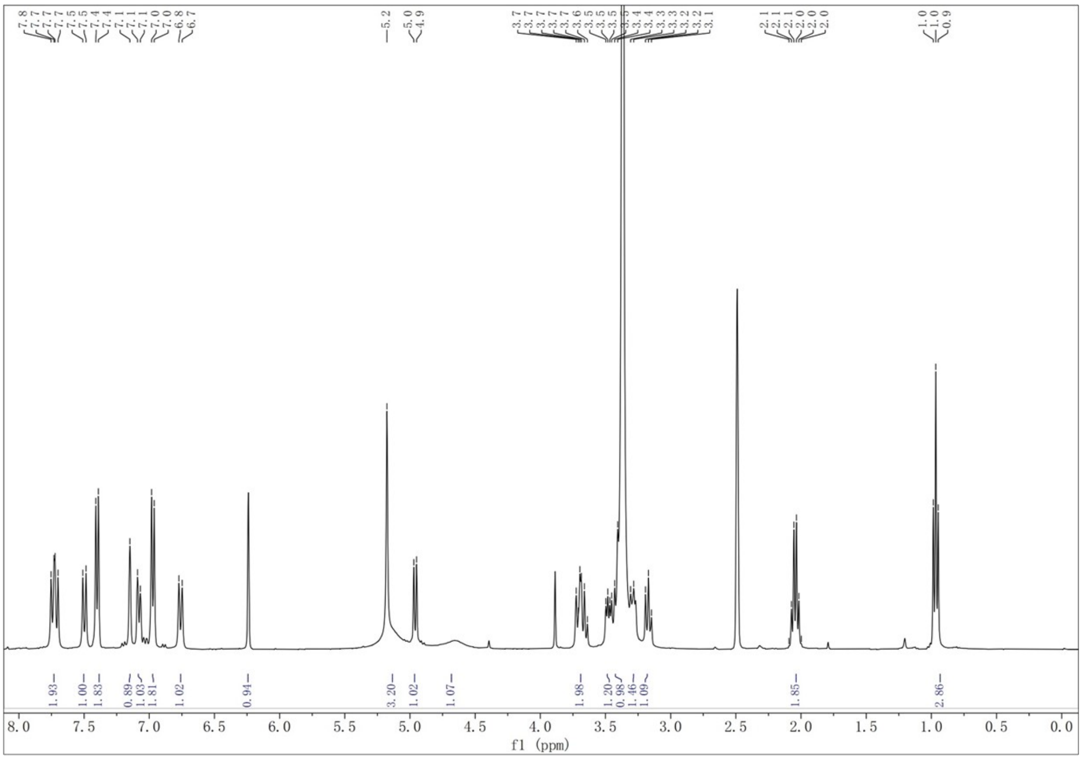

Figure S12. ^{1}H NMR of **GlcNPr-Bn-HHPO** in DMSO-d_6.

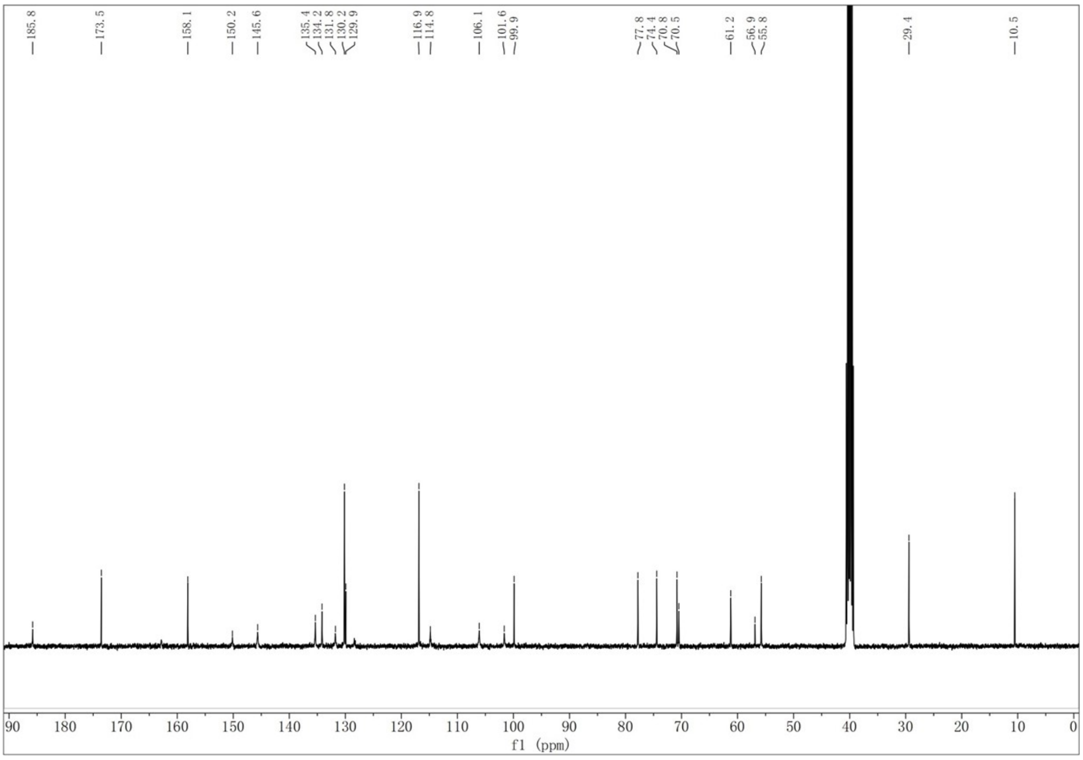

Figure S13. ^{13}C NMR of **GlcNPr-Bn-HHPO** in DMSO-d_6.

S4. Additional references

[1] Y.-H. Wu, G.-J. Wang, C. Guo, P.-P. Wang, J.-Y. Zhang, X.-L. Hu, Y. Zang, T. D. James, J. Li and X.-P. He, *Chem. Commun.*, 2024, **60**, 8240.

[2] F. Yan, X. G. Tian, Z. L. Luan, L. Feng, X. C. Ma and T. D. James. *Chem. Commun.*, 2019, **55**, 1955-1958.

[3] H. Jung, S. H. Park, W. H. Yang, J. W. Cho and I. Shin, *Sens. Actuat., B*, 2022, **367**, 132093.

[4] E. J. Kim, D. O. Kang, D. C. Love and J. A. Hanover, *Carbohydr. Res.*, 2006, **341**, 971-982.

[5] E. J. Kim, M. Perreira, C. J. Thomas and J. A. Hanover, *J. Am. Chem. Soc.*, 2006, **128**, 4234-4235.

[6] S. Cecioni and D. J. Vocadlo, *J. Am. Chem. Soc.*, 2017, **139**, 8392-8395.

[7] J. Y. Hyun, S. H. Park, C. W. Park, H. B. Kim, J. W. Cho and I. Shin, *Org. Lett.*, 2019, **21**, 4439-4442.

[8] J. Boo, J. Lee, Y. H. Kim, C. H. Lee, B. Ku and I. Shin, *Front. Chem.*, 2023, **11**, 1133018.
